# Investigation of Electrically-Assisted Rolling Process of Corrugated Surface Microstructure with T2 Copper Foil

**DOI:** 10.3390/ma12244144

**Published:** 2019-12-11

**Authors:** Shaoxi Xue, Chunju Wang, Pengyu Chen, Zhenhai Xu, Lidong Cheng, Bin Guo, Debin Shan

**Affiliations:** 1 National Key Laboratory for Precision Hot Processing of Metals, Harbin Institute of Technology, Harbin 150001, China; xueshaoxi@hit.edu.cn (S.X.); chenpengyu66@126.com (P.C.); xzhenhai@163.com (Z.X.); shandb@hit.edu.cn (D.S.); 2 Robotics and Microsystems Center, Soochow University, Suzhou 215131, China; ldcheng@suda.edu.cn

**Keywords:** surface microstructure, electrically-assisted rolling, current density, T2 copper foil

## Abstract

Electrically-assisted (EA) forming is a low-cost and high-efficiency method to enhance the formability of materials. In the study, EAF tensile tests are carried out to study the properties of T2 copper foil in an annealed state, and the effect of the electric current on the forming quality of corrugated foils is further studied in the EA rolling forming process. The result shows that the current reduces the flow stress and the fracture strain, which is different from the result of rolled samples. The joule heating effect on mechanical properties is significant in EA tension, and the softening effect of the surface layer can be observed at tensile strength, due to the grain size effect. Moreover, the current can weaken the grain size effect. In the rolling forming process, the influence of different electrical parameters on the forming height is remarkable, especially for the rolled T2 copper. The appropriate electrical parameters can improve the forming height, while keeping a small thickness thinning. Nevertheless, the high current density will lead to local rupture. This study proves that the current can improve the forming quality of the corrugated foils and is a promising surface texture forming process.

## 1. Introduction

Surface texture is highly critical in aviation, aerospace, and so forth, due to the special function of physics and chemicals, such as the drag reduction, the hydrophobicity, optics, heat and mass transfer [1]. The fabrication methods of the micro texture include electrochemical micromachining, laser surface texturing, electric discharge texturing, lithography, and micromachining. Electrical chemical micro machining is an advanced process which can fabricate the surface texture in metallic plates. Lee et al. [2] studied the effects of the inter-electrode gap, pulse rate, and electrolytic inflow velocity on the forming accuracy of the micro channel. A laser surface texturing to fabricate the surface texture was conducted by Tang et al [3]. They produced spikes on brass substrate, which had a super-hydrophobic function, using a pulse laser ablation process, and the height of these spikes related to the power of the laser beam. Electric discharge texturing can change the mechanical properties of the sample surface and form the micro structure by a high temperature [4]. Lithography involves multiple steps, like insulation, mask generation, and machining. He et al. [5] conducted lithography and an etching experiment to generate the micro-pillar-based surface textures on a silicon wafer and fabricate the nano pillars on the micro pillars. Hung and Lin [6] studied the micro scale tool piece electrode and the fabricating high-aspect-ratio micro channel on bipolar plates by electrical discharge machining. The surface texture can be easily fabricated by these methods mentioned above. However, laser surface texturing and electric discharge texturing can generate heat-affected zones or thermal residual stresses. Micromachining is not economically viable because of a lot of material waste. Electrochemical micromachining and lithography pose environmental concerns. A method which meets the demands of mass production and environmental protection is needed.

Rolling forming is a promising process for manufacturing surface microstructure sheets because of its low forming force, high efficiency, low cost, and good forming accuracy [7]. Ma et al. [8] found that the grain size and the crystal texture had a significant effect on the springback by rolling forming. Huang et al. [9] observed that the maximum thickness thinning of the micro channels with aspect ratios up to 1.0 was 18.7% by the micro-channel rolling forming test. Zhou et al. [10] improved the forming depth of the micro-structure and flatness of sheets by increasing the relative speed of the upper and lower rollers using a new desktop roll forming tool.

When the ratio of sheet thickness to grain size was small, the formability decreased [11]. The studies show that the current can reduce the flow stress, improve plasticity, and formability, this is called the electroplastic effect [12]. Ross et al. [13] presented the direct current (DC) EA compression experiments of Ti-6Al-4V alloy. The forming load was significantly reduced and the formability was improved. Andrawes et al. [14] found that the forming energy decreased, but the ductility and the elongation also decreased by the DC EA tension of aluminum alloy. However, the pulse current can not only reduce the flow stress but also significantly increase the elongation of aluminum alloy, according to the studies of Salandro et al. [15] and Roh et al. [16]. Jeong et al. [17] reported that the elongation significantly decreased when the pulse current was applied via the tensile test of trip-aided steel. They believed that the increase of the stability of retained austenite inhibited the effect of mechanically induced martensite transformation with the increase of temperature. In the investigation of Gennari et al. [18], the uniform elongation and total elongation were improved greatly in the EA tensile test, while the changes of the yield stress and ultimate tensile stress were inapparent, compared to the thermal test. Perkins et al. [19] conducted a series of studies by the EA compression of different metals, and found that there was a threshold current density in EA compression. Later on, Jones et al. [20] found that when the current density reached 30 A/mm^2^, the compressive properties of AZ31 magnesium alloys were remarkably improved without fracture. Siopis et al. [21] reported that with the increase of grain size, the degree of flow stress reduction became smaller, while the threshold value of the current density became larger. The decrease of the flow stress was clearer using a current when the samples underwent pre-plastic deformation [22,23]. Ross et al. [13] indicated that the thermal effect was not the main factor. The decrease of the grain size improved the degree of the joule heat rise and stress decrease, moreover, local intergranular voids and partial grain boundaries melted, according to the study of Fan et al. [24]. The effect of the current on the size effect was studied by Siopis et al. [21]. They believed that when the current was applied, the data dispersion would be reduced. Their studies indicated that the current can weaken the size effect [25,26]. Compared with the tensions at room temperature, oven-heated and air-cooled conditions, the fracture stresses were smallest and the fracture strains were largest in the EA tension [27].

Scholars also studied the EA forming process, such as drawing, rolling, bending, and embossing. Egea et al. [28] studied the current-assisted drawing process of 308L stainless steel. It was found that the material formability and energy efficiency increased by 11.9% and 7.6%, respectively. Li et al. [29] improved the toughness and strength of zirconium by EA rolling and subsequent low temperature annealing. Khal et al. [30] reported that with the increase of the current density, pulse frequency, and pulse duration, the springback can be further reduced by EA bending. Mai et al. [31] conducted the EA micro-channel embossing process of SS316L. When the current density increased to 50 A/mm^2^, the micro-channel depth increased to 26%. Cao et al. [32,33] studied the effects of the rolling force and the joule heat and friction on the forming quality of the microstructure by the EA roll forming process. The forming depth and width of the micro-channels increased with the increase of the temperature, rolling force, and friction force. It was proved that the joule heat was the main reason for the increase in the forming depth.

To conclude, the EA tension tensile and compression mechanical property have been sufficiently investigated. However, there are few studies on the EA process, especially on the thin sheet metal with microstructures. The influence of electrical parameters on the forming quality of the microstructures is not clear. In this paper, the EA uniaxial tension of T2 copper is studied experimentally. The effects of different current parameters on the flow stress, fracture strain, and grain size effect are analyzed. Furthermore, the EA rolling forming process is investigated, and the results of different electrical parameters on the forming quality are analyzed, which verify the feasibility of the EA rolling forming process.

## 2. Materials and Methods

### 2.1. Experimental Materials

**Figure A1:**
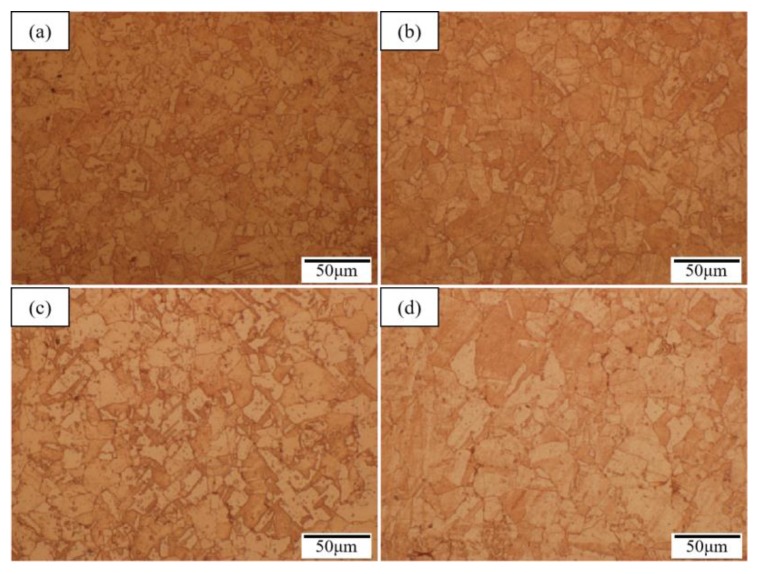
Microstructure of T2 copper foil along the thickness direction after annealing treatment:(**a**) 350 °C; (**b**) 450 °C; (**c**) 550 °C; (**d**) 650 °C.

**Figure A2:**
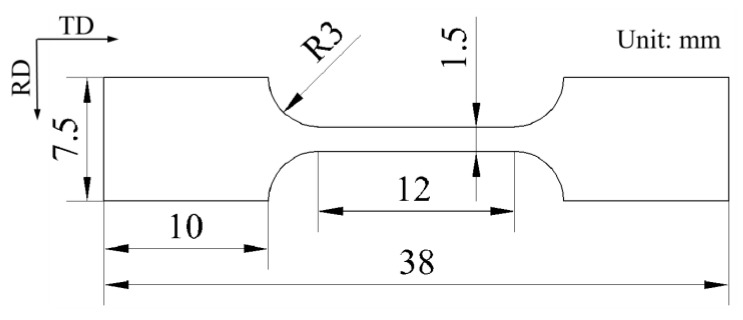
Geometry of tensile samples.

In this work, commercial T2 copper foils with the thickness of 100 μm in the rolled state and annealed state (annealed at 310 °C and air cooled) were chosen as the experimental materials. To study the grain size effect on the deformation behavior, T2 copper foils in the rolled state were annealed at the temperature of 350, 450, 550, and 650 °C for 1 h to obtain different grain sizes. The microstructures were examined using optical microscope (OM) after grinding, polishing, and etching. The microstructure and obtained grain size are given in Figure A1 and Table 1, respectively. The T2 copper foil was machined to tensile test samples by the electrical discharge machining (EDM) method, as shown in Figure A2.

### 2.2. Experimental Set-Up

**Figure A3:**
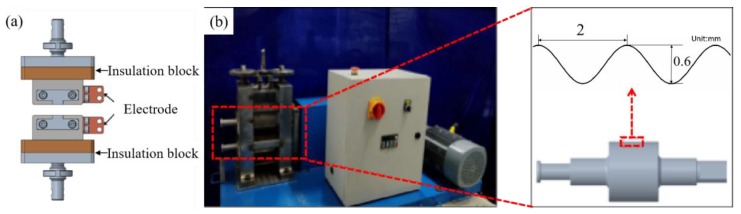
EA device: (**a**) tensile insulated grip; (**b**) EA rolling device.

The EA micro-tension device was developed on the basis of a tension testing machine (CMT8502). Two insulation blocks were inserted between the EA tension grips and tension testing machine, as illustrated in Figure A3a. As shown in Figure A3b, the EA rolling system and corrugated surface microstructure were designed to conduct the EA rolling experiment. The power supply (JX-HC DC pulse power, Lanzhou, China) had a maximum voltage of 30 V, which could output a high frequency square wave pulse current, as shown in Table 2. The temperature of specimens was measured by a FLIR infrared camera.

## 3. Results and Discussion

### 3.1. EA Uniaxial Tensile Test

**Figure 1:**
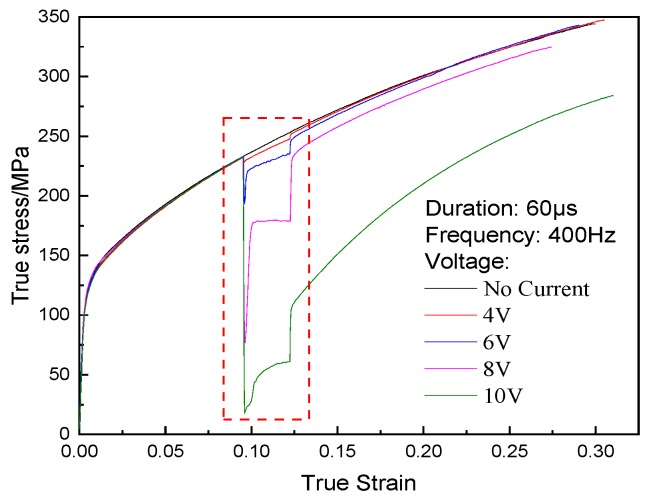
True stress–strain curves of annealed T2 copper.

EA uniaxial tensile tests using samples of annealed state were carried out under a strain rate of 10^−3^s^−1^ at room temperature. The current started and continued 30 s when the tensile test was conducted for 100 s. It is found that the flow stress decreases nearly instantly as soon as the electric current is present, and the flow stress decreases with the increase of voltage, as shown in Figure 1. The flow stress increases after closing the power, but compared with the uniaxial tension of no current, the flow stress is still lower, especially at the great voltage.

**Figure 2:**
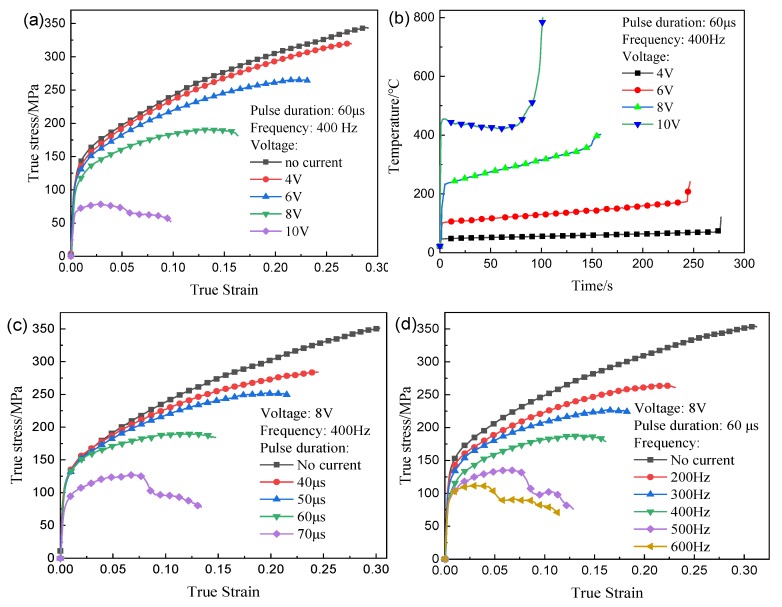
EA tensile test results: (**a**) different voltages; (**b**) temperature of gauge; (**c**) different durations; (**d**) different frequencies.

To further study the effect of the current on mechanical properties, the current was present during the tension test. The influences of voltage, frequency, and pulse duration on the mechanical properties and temperature are shown in Figure 2. It is seen from Figure 2a that the flow stress and the fracture strain decrease with the increase of the voltage in the EA tension. The result is similar to that of Zhang et al. [27]. They conclude that the effect of the joule heating on the mechanical properties can be neglected. Figure 2b presents the maximum temperature of the specimen in the gauge section. The temperature increases instantly by joule heating and then changes slowly for the balance of the joule heating and air cooling until the fracture of samples. Furthermore, as the the voltage increases, the temperature increases because of the Joule heating effect. Mean temperatures are 60.35 °C at 4 V, 138.25 °C at 6 V, 301.45 °C at 8 V, and 447.00 °C at 10 V, respectively. When the voltage increases from 4 to 10 V, the temperature increases from 60.35 to 447.00 °C, the reduction rate of the tensile strength increases from 15.68% to 77.24%. The maximum reduction rate (77.24%) is much larger than that (23%) in the reference [27]. It needs to be noted that the temperature rise is significant in this study, and maybe the joule heating effect is the main reason for the change of mechanical properties.

Figure 2c,d depicts the variations in the true stress–strain for various pulse durations and frequencies, respectively. It can be seen that the flow stress and fracture strain decrease as the pulse duration or frequency increases. Note that the flow stress continues to decrease, but a change of the fracture strain is not apparent, which indicates that the high frequency is conducive to the plastic deformation.

From Figure 2, it is found that when the temperature is high, such as 447 °C, the flow stress reduction remarkably increases, which indicates that the current density (related to the voltage, frequency, and pulse duration) threshold is mainly temperature-independent.

It is interesting that the results are different from that of rolled samples. The flow stress drop is more remarkable, moreover, the fracture strain increases significantly in the EA tension test of T2 copper foil (rolled state) [34]. The current promotes dislocation movement, makes dislocation easier to overcome obstacles, and restrains the slip band generation. Hence, it can be understood, from the results, that the current has a more significant influence on the samples of higher dislocation density.

**Figure 3:**
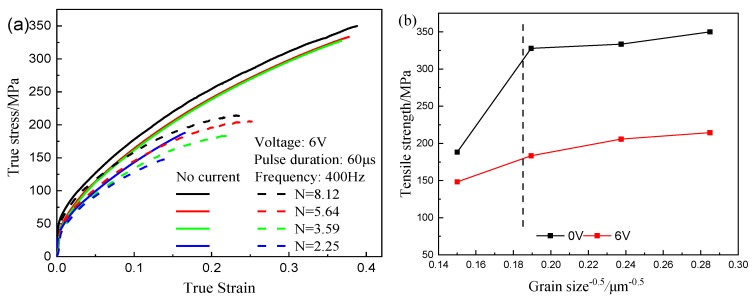
Tensile test results with different *N* value: (**a**) EA tensile test; (**b**) flow stress and fracture strain.

In order to investigate the grain size effect on the EA tension, the EA tensile tests of various grain sizes and *N* values were carried out, as shown in Figure 3. Figure 3a shows that the flow stress and fracture strain decrease in the EA tension, due to the increase in the grain sizes, which corresponds to results in Figure 2a. The relationship between the tensile strength and grain sizes at a voltage of 6 V is depicted in Figure 3b. This cannot be interpreted by the Hall–Patch relation, due to the softening effect of the free surface at *d* = 44.49 μm [35]. This is due to the interactive effect of specimen and grain sizes. However, the softening effect is weakened when the current is present. This result indicates that the current can weaken the grain size effect, which corresponds to the result of Wang et al [25].

### 3.2. Rolling Forming of Corrugated Surface Microstructure

**Figure 4:**
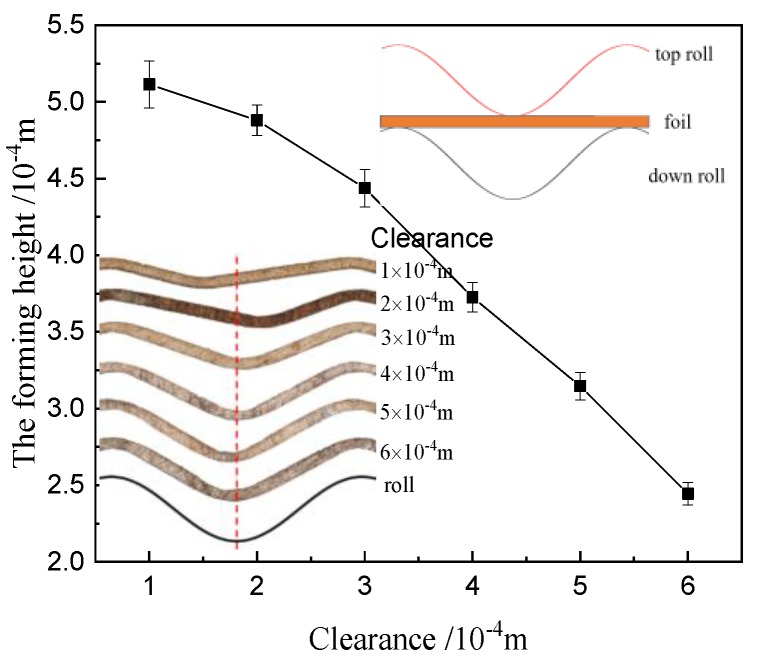
Forming height with different clearances.

**Figure 5:**
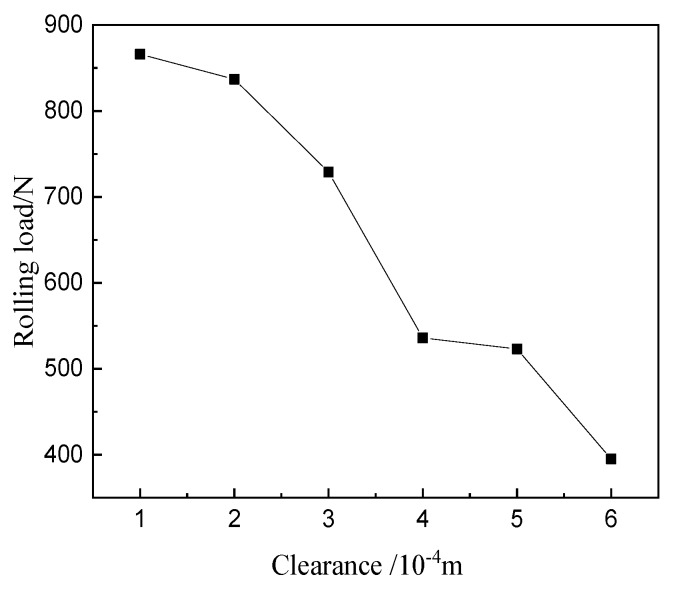
Rolling load with different clearances.

The rolling tests were conducted to investigate the effect of clearance between the upper roller and lower roller on the formability of the surface microstructure. The clearance is relative to the initial position in Figure 4. Figure 4 and Figure 5 show the forming height and the rolling load by the load sensor (Interface MSC-130KN-375, Shenzhen, China) under different clearances. The rolling load and the forming height increase with the decrease of clearance.

**Figure 6:**
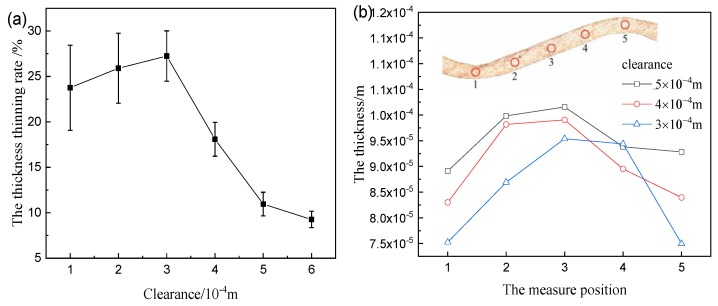
Thickness thinning measure results: (**a**) maximum thickness thinning rate; (**b**) distribution of thickness.

**Figure 7:**
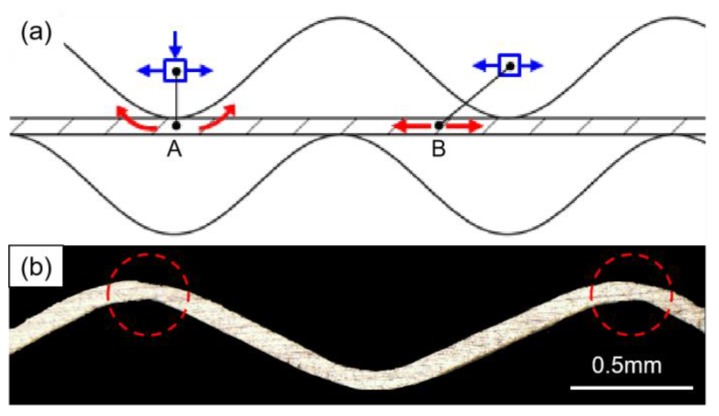
Mechanical analysis in rolling process: (**a**) scheme of mechanical analysis; (**b**) thickness thinning.

As the clearance decreases, the maximum thickness thinning rate and data dispersion increase, as shown in Figure 6a. The thicknesses of the wave crest and trough are at a minimum (in Figure 6b). Figure 7a presents the scheme of mechanical analysis. The bending and tensile deformation occur at part A, but the tensile deformation is only at part B, moreover, the tensile stresses are similar at part A and part B. But part A has a different bending deformation that results in the large deformation and thickness thinning (Figure 7b).

### 3.3. EA Rolling Forming

**Figure 8:**
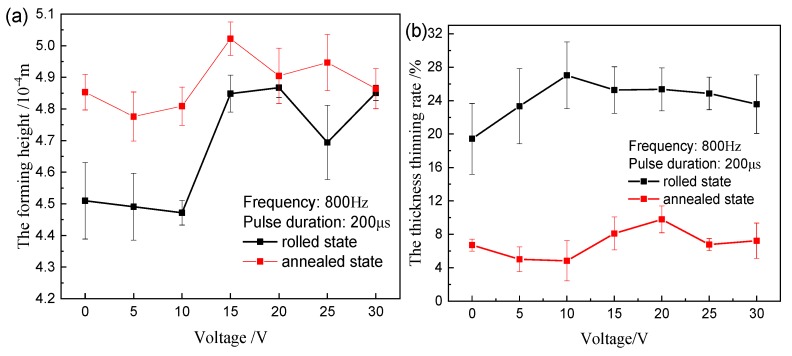
Experimental results of different voltage: (**a**) forming height; (**b**) maximum thickness thinning rate.

**Figure 9:**
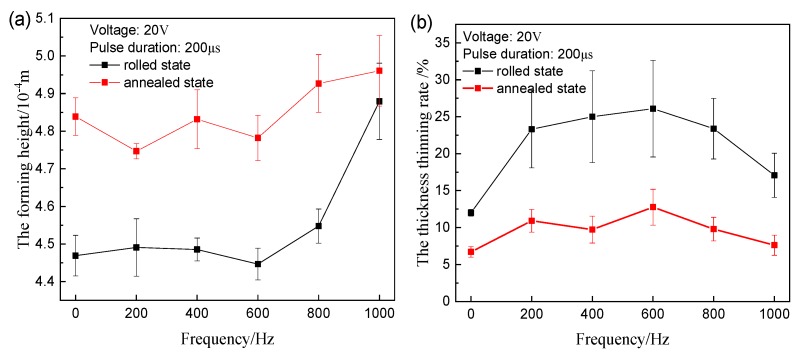
Experimental results of different frequency: (**a**) forming height; (**b**) maximum thickness thinning rate.

**Figure 10:**
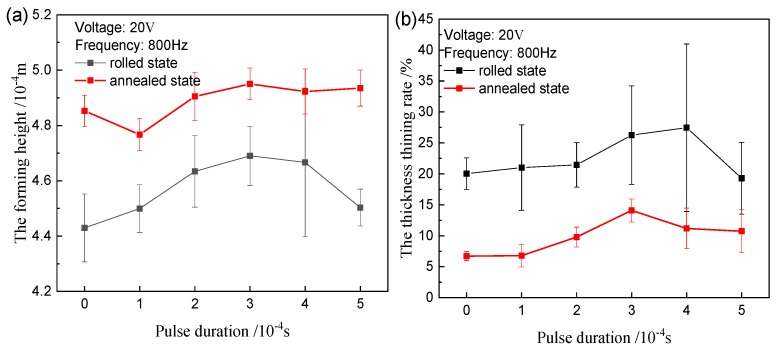
Experimental results of different duration: (**a**) forming height; (**b**) maximum thickness thinning rate.

The EA rolling tests were conducted at a roll speed of 0.75 r/min and a clearance of 0.4 mm at room temperature. The influences of various voltages, frequencies, and pulse durations on the forming height and the thickness thinning rate are demonstrated in Figure 8, Figure 9, and Figure 10, respectively. It can be seen from Figure 8a that the height has a significant improvement at 15 V for two kinds of materials, especially the rolled samples, which is attributed to the flow stress drop due to the joule heating effect.

The thickness thinning rate of annealed samples is significantly lower than that of rolled samples, as shown in Figure 8b. But the difference between various voltages is insignificant, whither in a rolled or annealed state. A small part of the wave crest and trough crack for the rolled samples is due to the higher temperature when the voltage increases to 20 V, but this is not the case for the annealed samples.

It is observed from Figure 9a that the height remarkably increases at 800 Hz for two kinds of materials. The difference of the thickness thinning rate between various frequencies is also not significant. However, the higher frequency can not only increase the forming height, but also reduces the thickness thinning, as shown in Figure 9b.

It is observed from Figure 10a that as the pulse duration increases, the forming height increases for two materials. Compared to the effect of the voltage and frequency, the change of height is smaller. Meanwhile, the thickness thinning rates also differ slightly (Figure 10b). For the high pulse duration (300 µs), some cracks also are observed for two state samples.

To summarize, when the current density threshold is reached, the joule heating temperature is sufficiently high to reduce the flow stress remarkably, there is a range of current density, which can significantly improve the forming height and maintain a smaller thickness thinning rate. Nevertheless, a larger current density can lead to a local fracture.

## 4. Conclusions

In this work, the effects of voltage, frequency, and pulse duration on tensile properties and rolling forming process of T2 copper are investigated by the EA tension and EA rolling process. The influence of the current on the grain size effect is studied by EA tension with T2 copper foil of various grain sizes. The following results can be concluded:

(1) The flow stress and fracture strain decrease as the current density increases, which is mainly attributed to the joule heating effect.
(2) The softening effect of the surface layer is significant, and this is due to the grain size effect at coarse grains. However, it can be weakened in the EA tension.
(3) With the decrease of clearance, the forming load and the forming height of the microstructures increase gradually, and the thinning of the wave peaks and troughs is more serious than that of other parts.
(4) The forming height significantly increases by the EA rolling forming process, moreover, the wall thickness change slightly under proper current parameters. Thus, it is conducive to the formation of corrugated foils.

## Figures and Tables

**Table 1:** Grain sizes under different temperatures.

Temperature (°C)	350	450	550	650
Grain size *d* (10^−6^m)	12.31	17.73	27.87	44.49

**Table 2:** DC power parameters.

Voltage/V	Frequency/Hz	Pulse Duration/10^−6^ s
0~30	10~1000	3~500
